# Hemorrhagic Spinal Schwannoma in Thoracolumbar Area with Total Paraplegia

**DOI:** 10.1155/2019/7190739

**Published:** 2019-01-02

**Authors:** Ahmad Jabir Rahyussalim, Rizky Priambodo Wisnubaroto, Tri Kurniawati, Alfariq Senja Belantara Latsarizul, Nuryati Chairani

**Affiliations:** ^1^Department of Orthopaedic and Traumatology, Faculty of Medicine, Universitas Indonesia-Cipto Mangunkusumo Hospital, Jakarta, Indonesia; ^2^Stem Cell Integrated Medical Service Unit Cipto Mangunkusumo Hospital, Faculty of Medicine, Universitas Indonesia, Stem Cell and Tissue Engineering Research Center, IMERI Universitas, Jakarta, Indonesia; ^3^Department of Anatomical Pathology, Faculty of Medicine, Universitas Indonesia, Cipto Mangunkusumo Hospital, Jakarta, Indonesia

## Abstract

Hemorrhagic schwannoma in the medulla spinalis is a rare occurrence. It is a variant of the slow-growing nerve sheath tumor that usually has subtle clinical symptoms. Injury to the spinal schwannoma that was previously suspected by spinal manipulations may accelerate the progression of symptoms and cause an acute presentation of paraplegia. We report a case of a patient that was suspected of an intradural tumor with paraparesis that initially refused treatment. Spinal manipulation procedures were performed outside of the hospital setting with subsequent advancement of paraparesis. A surgical intervention was performed, which found that the tumor mass has grown along with hemorrhage within the schwannoma. The bleeding within the mass may have caused the acute paraplegia that is rarely reported. The patient had a fair improvement on her lower motor extremity function from 1‐2 to 3‐4 out of 5 at six‐month follow-up.

## 1. Introduction

Schwannoma tumor in the medulla spinalis is a relatively common tumor. Every year, worldwide, the number of reported cases in primary spinal neoplasms is around 1.3 to 10 for every 100,000 cases [1]. Spinal nerve tumor can be divided according to its location: extradural, intradural, or both. Agewise, this tumor has no predisposition between males and females. The patients may present themselves with a progressive neurological deficit which correlates with the position of the tumor, usually at the cervical or lumbar regions [1–3].

Hemorrhagic type of schwannoma is very rare, with only a few cases reported previously. Acute neurological deficit may be caused by intratumoral bleeding which causes cord compression. This has been noted in cranial schwannomas previously, but in a spinal schwannoma, this instance is seldom found [4].

We are presenting a report of a female with a case of hemorrhagic type of thoracolumbar schwannoma with sudden paraplegia, which was treated surgically after a period of therapy refusal and spinal manipulation by a traditional bone setter. A brief review of the literature is included to discuss current management options for these cases.

## 2. Case Illustration

**Figure 1:**
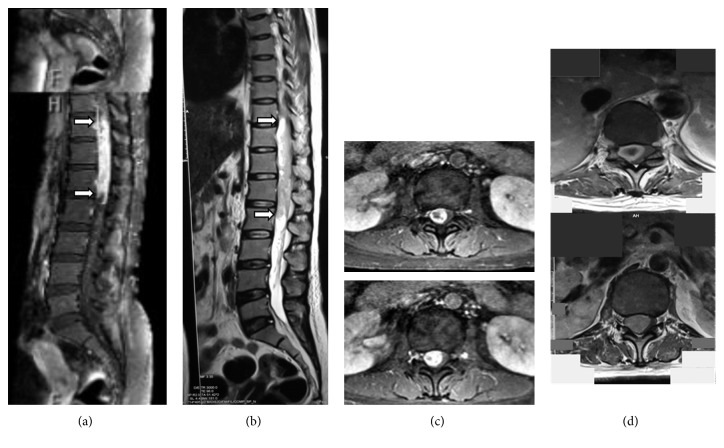
Thoracolumbar T2W1 MRI in the sagittal plane (a and b) and axial plane (c and d) at the tumor height. We can observe the growth of hyperintensity from T10 to L2 in (b) compared with the initial MRI taken (a). Tumor margins are marked with the arrows. Axial T2W1 images on the tumor edges are shown in (c) and (d); the tumor diameter and length have increased in the space of four months.

A 38-year-old female patient came with a chief complaint of difficulty standing up from squatting position since 2 years ago. The patient also suffered heaviness and numbness from her hips that radiated to both of her knees and ankles. The symptoms worsened steadily in the past 4 months with both lower limbs getting weaker. Laboratory results came out normal, with no signs of infection or positive tumor markers. Radiological examination showed no apparent abnormalities as well. An MRI was obtained, and a tumor mass in the intradural region level of T10–T12 was found (Figure 1). At that time, the patient was offered surgery, but she chose to undergo treatment with a bone setter. Around two months later, the patient returned to hospital with profound weakness on her lower extremity. Her physical examination revealed paresis from her thigh on both lower extremities grade 1-2/5 power in left and right lower limbs, respectively. Increased patellar reflexes were found on both limbs. Another MRI was performed and showed that the mass had grown to lumbar vertebrae L2, accompanied with worsening of the neurological statuses and impaired sensibility, as well as defecating and urinating problems (Figure 1). From the history, spinal manipulation procedure was performed by a bone setter, although no specific techniques were available for review.

**Figure 2:**
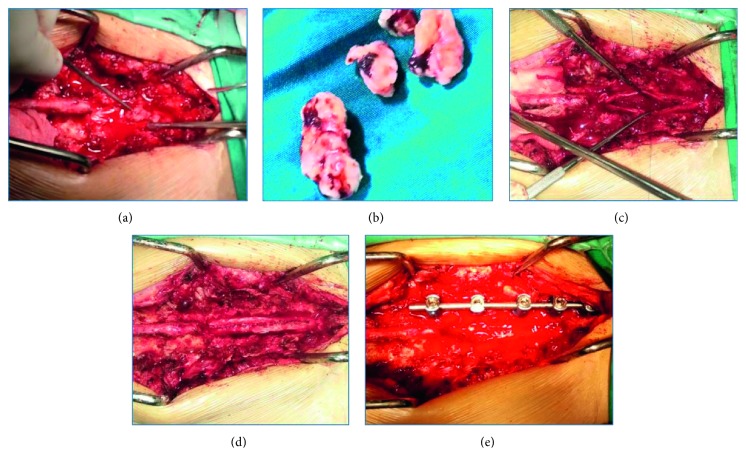
Intraoperative pictures: the exposure of the spine using a posterior approach. Durotomy was performed from thoracal T10 to lumbar L1. The intradural tumor mass can be seen in (a). Tumor was then exsanguinated and evacuated. The tumor specimens were covered in blood and rich in vessels (b). We can see the empty space in dura, which was previously filled with the tumor. Dura was then closed with polypropylene suture 6.0 (c and d). The pedicle screw and rod system was implanted only on the left side to strengthen the spine after laminectomy in four levels.

A surgical procedure was proposed for exploration and decompression to the patient. The operation started by opening the lamina on T10–T12 levels, followed with laminectomy and hemostatic procedure to stop the bleeding, until the dura was exposed (Figure 2). A dense mass from T10 to T12 was palpable from the dura layer. After we exposed the lamina, we observed that the dura was tense from touch, and a solid mass underneath the dura was palpable from T10 to L2. Intradural tumor excision was performed by a sharp 3 mm incision in the midline of the dura and then continued with blunt dissection, opening the dura layer to caudal L2 and to cranial T10. Dura was then parted with stay thread until the vessel-rich tumor mass was exposed. The tumor mass was excised, and the surgical field was contaminated by blood from hematomas from tumor vessels (Figure 2). Bleeding was found to originate from the anterior part and posterior part of the cord, no bleeding source from the cerebrospinal fluid nor the subarachnoid space. The tumor mass was successfully evacuated with fragments of hematomas and necrotic tissues. The dura layer was then closed with a continuous suture. The tumor mass was fixed and transported for histopathology examination (Figure 2).

At the time of discharge, the patient did not regain the function on her lower extremities (1-2 out of 5 on neurological motor examination). After six months of follow-up, some improvement on her lower extremity function was noted. Motor strength was returned to 3-4 out of 5, and the patient was able to ambulate using a walker. No improvement of her bowel and bladder symptoms was noted.

## 3. Hemorrhagic Type of Spinal Schwannoma

Hemorrhagic type of spinal schwannoma is a rare case and more commonly found in the cranial region of the spine [5]. The tumor in the spine is more likely found around the thoracolumbar junction region or the cervical region due to the high movement in these segments and the high density of the nerve roots [2–5]. The difference between this type of schwannoma and other types is the clinical finding of bleeding in the tumor mass intraoperatively. From microscopic findings, it is shown that the hemorrhagic background along with Verocay bodies, spindle-shaped Schwann cell areas (Antoni type A tissue), and loose myxoid area (Antoni type B tissue) [5–8].

The hemorrhagic type of spinal schwannoma is a slow-growing benign lesion and occurs mostly as a solitary mass which may occur throughout the spinal canal [8–10]. Only 4% of patients are present with multiple lesions. The cells are derived from Schwann cells of the dorsal nerve roots, and only 23% of cases may arise from the ventral nerve roots. They are encapsulated neoplasm, firm, and hemorrhagic. They are mostly classified as intramedullary and extramedullary tumors [8–10].

Bleeding from hemorrhagic schwannomas is thought to arise from main theories, vascular and mechanical theory. Vascular theory postulates that spontaneous thrombosis occurred with distal tumor necrosis and caused secondary bleeding. The tumor maybe obliterated by endothelial proliferation with recanalization, whereas mechanical theory argued that movements of the spine induce forces on tumor vessels resulting in extravasation of blood [10].

Diagnosis of hemorrhagic type of spinal schwannoma is made according to clinical history, physical examination, laboratory examination, radiological examination, and histopathological examination. Patients initially presented with local pain and signs of compression of neural structures with neurological deficits developing in late phase [5, 8]. Patients feel segmental pain followed by local pain, motor weakness, gait ataxia, bladder paresis, and dysesthesia. Spinal schwannomas are generally slow-growing, and the symptoms worsen over time. In physical examination, we can find motoric or sensory impairment (neurological deficit) [5, 8]. In laboratory examination, we can find no signs of infection and positive/negative tumor markers. In radiological examination, MRI is used to evaluate the location and the extent of the schwannoma. It is important to evaluate the degree of extraspinal extension of the tumor for selecting the best surgical approaches and the relationship of the tumor and the major vessels, including vertebral artery and abdominal vascularization. The characteristics of the hemorrhagic-type spinal schwannoma are the isointensity in T1 and hypointensity in T2 MRI images. Inhomogeneous or absent enhancement usually points to bleeding, showing hypointense image in T1 with hyperintensity in T2 weighted images [5, 8, 9]. In histopathology examination, we can find bleeding in the mass, Verocay body, cellular area (Antoni A), and loose myxoid area (Antoni B) [5, 9].

Early surgical removal is the treatment of choice, and complete removal can be achieved in almost all cases with good outcome in majority of cases [2, 5, 11]. Spinal schwannomas are usually present eccentrically and dorsolateral to the spinal cord. After the dura is opened, accessible via posterior or posterolateral approach, the schwannomas are easily seen. Other approaches may be done depending on the location of the tumor. After the schwannomas are exposed, the dissection-plane on the tumor surface must be identified, and the dura must be incised to reflect off the tumor surface. The tumor and its capsule are excised by cauterizing the surrounding vessels, and the tumor must be evacuated and then histopathology examination performed. Postlaminectomy instability and deformity may occur especially after multilevel laminectomy, and posterior stabilization can be performed as necessary [10].

## 4. Discussion

Hemorrhagic-type spinal schwannoma is uncommon, with only 13 cases ever being reported up to date. Prevalence among male and female patients is 7 : 6, with an average age of patients around 48.38 years old. From the 13 reported cases, hemorrhagic type of schwannoma was found in C6-C7 (30.77%), T9-T12 (53.85%), L1-L2 (7.6%), and T12-L1 (7.6%). Schwannoma is usually intradural-extradural (58%), extradural (27%), dumbbell-shaped intradural-extradural (15%), and intradural (less than 1%). In our case, tumor was found intradural on the T10–T12 level, and the mass had grown to reach all the way to lumbar vertebrae L2 in just 4 months, making this case a case of hemorrhagic spinal schwannoma and also a long segmental hemorrhagic type of spinal schwannoma [5, 6].

Hemorrhagic type of spinal schwannoma is also uncommon in hemorrhagic neoplasm cases. Neoplasms accompanied with hemorrhage are usually cavernomas, ependymomas, or hemangioblastomas. Hemorrhage in the spine is usually due to coagulopathy, malformations, or trauma. In our case, hemorrhagic type of spinal schwannoma as a rare case in hemorrhagic neoplasm may occur because of mechanical factor after manipulation by the bone setter [10].

Clinical symptoms vary depending on the size and location of the tumor. Hemorrhagic type of schwannoma usually has an acute onset with clinical manifestation of segmental pain followed by radicular pain and motoric impairment. Some of the clinical symptoms ever recorded from the 13 reported cases are flaccid paraplegia, spastic paraplegia, urine retention, urine incontinence, progressive weakness, upper limb pain, cervical pain, cervical myelopathy, dysesthesia, back pain, severe paraparesis, nausea, and limb pain. It is very rare to have an acute and urgent onset. In our case, the first clinical manifestation was difficulty standing up from squatting position and heaviness and numbness in the hip that radiated to the knees and ankle. Progressive motoric impairment presented on both limbs within 4 months, accompanied with worsening of neurological statuses, impaired sensibility, as well as defecating and urinating problems.

In our case, the spinal manipulation therapy exerted by the bone setter may be the cause of the rapid progression of the symptoms. Previous reports by Cohen and Uemura et al. described the case of acute paraplegia or paresis following a minor thoracic injury or physical exercise in patients that were later found to have intradural tumors, although further studies are needed to fully comprehend the mechanism [6, 7, 9, 11–16].

Hdeib et al. in their paper cited a review of epidural hematomas following spinal manipulation therapies. In the review, some cases reported spinal epidural hematomas directly after manipulation. The literature however did not include the number of spinal intradural hematomas following the manipulation.

**Figure 3:**
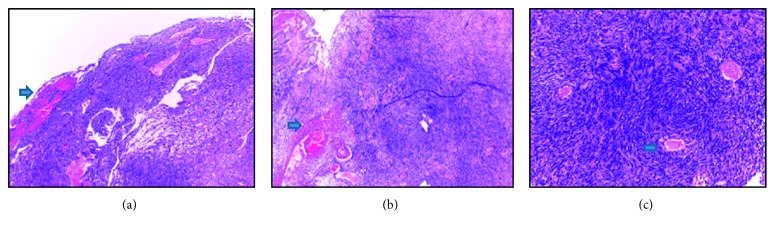
Histopathology results with 4x magnification. The green arrows show tumor vessels (a) and bleeding tumor mass with well-defined edge (b). Palisade cells (Antoni A cells), hypocellular (Antoni B cells), hypercellular, and hemorrhage areas (c).

The most important diagnostic examination in hemorrhagic type of schwannoma is MRI and histopathology (Figure 3). Another essential examination is laboratory tests where the result of this case came out normal with no signs of infection or positive tumor markers. The characteristics of hemorrhagic type of spinal schwannoma in our case are isointensity in T1 and hypointensity in T2, as well as inhomogeneous or absent enhancement, which define acute bleeding. However, other findings are also possible, such as hypointensity in T1, hyperintensity in T2, and homogenous enhancement as commonly found in spinal schwannoma. In our case, we can clearly see the intradural tumor mass on the T10–T12 level with hyperintensity in T1 and T2 accompanied with absent enhancement. This might be due to fat and blood, which may appear as hyperintensity in T1 and T2.

A surgical procedure was performed to our case on the T10–T12 level, and reddish vessel-rich tumor mass was found. The whole tumor mass was then evacuated and sent for histopathology examination. From microscopic findings, the following were observed: bleeding in the mass, Verocay body, cellular area (Antoni A), and loose myxoid area (Antoni B) (Figure 3). These findings conform with the characteristics of hemorrhagic type of spinal schwannoma.

The main procedure performed to the patients with hemorrhagic type of schwannoma or other spinal tumors is surgery (laminectomy), excision of the tumor as soon as possible [5, 9, 11, 14, 15]. For most cases with laminectomy, excision of the tumor gives improvement postoperative outcomes in neurological condition [9, 15]. Delay in operation raises the difficulties in restoring the neurological function after surgery, especially in cases with extensive tumor involvement [6].

## 5. Conclusion

A spinal schwannoma is a rare tumor that can present itself with a period of acute progression of symptoms. Spinal manipulation may aggravate the symptoms of the tumor and elicit bleeding of the tumor. An immediate surgical intervention to evacuate the tumor mass may improve the prognosis for these patients.
